# A Geostatistical Approach to Assess the Spatial Association between Indoor Radon Concentration, Geological Features and Building Characteristics: The Case of Lombardy, Northern Italy

**DOI:** 10.3390/ijerph8051420

**Published:** 2011-05-06

**Authors:** Riccardo Borgoni, Valeria Tritto, Carlo Bigliotto, Daniela de Bartolo

**Affiliations:** 1 Department of Statistics, University of Milan-Bicocca, Via Bicocca degli Arcimboldi 8, 20126 Milan, Italy; E-Mail: v.tritto@campus.unimib.it; 2 Agenzia Regionale per la Prevenzione e Protezione Ambientale del Veneto—Department of Padova, Via Ospedale 22, 35121 Padova, Italy; E-Mail: cbigliotto@arpa.veneto.it; 3 Agenzia Regionale per la Protezione dell’Ambiente della Lombardia—Central Department, Viale Restelli 3/1, 20124 Milano, Italy; E-Mail: d.debartolo@arpalombardia.it

**Keywords:** radon, geostatistics, kriging with external drift, IRWGLS, geological class, building factors

## Abstract

Radon is a natural gas known to be the main contributor to natural background radiation exposure and second to smoking, a major leading cause of lung cancer. The main source of radon is the soil, but the gas can enter buildings in many different ways and reach high indoor concentrations. Monitoring surveys have been promoted in many countries in order to assess the exposure of people to radon. In this paper, two complementary aspects are investigated. Firstly, we mapped indoor radon concentration in a large and inhomogeneous region using a geostatistical approach which borrows strength from the geologic nature of the soil. Secondly, knowing that geologic and anthropogenic factors, such as building characteristics, can foster the gas to flow into a building or protect against this, we evaluated these effects through a multiple regression model which takes into account the spatial correlation of the data. This allows us to rank different building typologies, identified by architectonic and geological characteristics, according to their proneness to radon. Our results suggest the opportunity to differentiate construction requirements in a large and inhomogeneous area, as the one considered in this paper, according to different places and provide a method to identify those dwellings which should be monitored more carefully.

## 1. Introduction

Radon (the ^222^Rn isotope, with a half-life of 3.8 days) is a naturally occurring decay product of uranium commonly found in rocks and soils. It is an odourless, colourless and tasteless gas that drifts upward through the ground to the Earth’s surface, undetectable to humans except by means of specialized measurement devices. Radon activity concentration is generally measured in Bq/m^3^.

Radon is known to be the main contributor to natural background radiation exposure. From epidemiologic studies, it was established that the major health risk related to radon and radon progeny exposure is lung cancer. In fact, radon is considered to be a major leading cause of lung cancer, second only to smoking. IARC (International Agency for Research on Cancer) has classified radon as a group 1 substance, that is to say, a substance with “sufficient evidence of carcinogenicity in humans” [1]. The American Environmental Protection Agency (EPA) estimates that 7,000 to 30,000 annual lung cancer deaths in the USA are caused by exposure to residential radon. For this reason, monitoring surveys have been promoted in a number of Western European countries in order to assess the exposure of people to this radioactive gas and to identify areas more prone to high indoor radon concentration (IRC) [2].

The main source of radon gas is the soil [3]. Radon can enter buildings through cracks or holes in the foundations and concrete floors and can reach high levels of indoor concentrations. Air pressure differences between the soil and the house can cause the soil air to flow towards the foundation of a building. Soil porosity and permeability can also affect IRC. For this reason IRC is expected to show regularities when monitored in space over a large region. Drawing maps to identify areas more exposed to IRC has been customary for a long time. Many of those maps are based on geological and geophysical reasoning and areas are classified according to soil and geological maps [4–6]. Geostatistical techniques have been introduced more recently. Kriging-like procedures [7] have been proposed for this task; see amongst others [8–11]. As it has been empirically found that IRC tends to show a log-normal distribution [12], many of those studies adopted a log-gaussian kriging to produce maps through punctual predictions on fine grids which discretised the region of interest. More robust kriging algorithms to distributional assumptions have also been suggested [13] and kriging procedures have also been employed to areal prediction [14]. Some authors suggested using hierarchical modelling for punctual predictions of radon concentration [15,16]. Growing interest in the geostatistical approach to map IRC is highlighted by a number of recent workshops on this topic such as the Annual Meeting of the International Association of Mathematical Geology held in Liègi in 2006 and the 8th International Workshop on the Geological Aspects of Radon Risk Mapping held in Prague in 2006.

However, IRC can largely depend on building characteristics such as building materials, ventilation and water supply that affect the entry of radon into the buildings and movement between rooms therein, as well as on the permeability and lithologic nature of the ground. The dependence of IRC on building and geologic factors has previously been studied in various papers. Statistical associations between radon measurements and housing factors as well as geologic indicators of high radon potential have been investigated in Canada [17], USA [15,16,18,19] and Europe [11,20–26].

In this paper, we aimed at two different but complementary objectives. Firstly we produced a map of IRC on a large region. Specifically, we considered the Lombardy region which is a wide area located in northern Italy. Covering a surface area of 23,800 km^2^, Lombardy is the fourth largest and most populated Italian region, with an estimated 9,800,000 inhabitants at the end of 2010, *i.e.*, about 20% of the whole Italian population. Furthermore, the Lombardy region has the second highest population density (about 412 inhabitants per km^2^) in Italy and one of the largest in Europe at the Eurostat NUT2 level [27]. The Lombardy territory is quite heterogeneous as it will be made clear later on in this paper, with about 40% of the whole territory being mountainous (Alpine and pre-Alpine region), whereas 47% is flat (the Padana Plain). Furthermore, the results of the national survey [28] showed that the mean IRC in Lombardy (116 Bq/m^3^) was quite higher than the national mean value (70 Bq/m^3^). The data of the Lombardy regional survey, considered in this study, confirm even higher concentration levels. Hence, assessing the population exposure at IRC and how it varies in space is a relevant environmental and health-related issue. For this goal, we used georeferenced data collected within an indoor radon gas monitoring survey conducted by the Agency of Environmental Protection of the Lombardy Region in 2003. Papers concerning IRC mapping in the Lombardy plain have previously been published (see for example [29]). However, these papers focused on a different approach based on geological arguments and used different and quite smaller datasets. Since geological information on the ground is potentially relevant for constructing a reliable map, we linked our data to external spatial (*i.e.*, georeferenced) databases containing soil and lithological information by a GIS exercise and obtained maps of IRC via a kriging with external drift (KED) algorithm.

Secondly we jointly analyzed the effect of geological and building characteristics through a multiple regression model. In order to account for the spatial correlation of the data we employed an iteratively reweighted generalized least squares (IRWGLS) algorithm. The model is extended semi-parametrically, through a B-spline, to account for a potentially non-linear effect of some covariates.

Finally we mention that soil gas radon can be a powerful parameter to improve IRC mapping and to identify areas more prone to high IRC, as it has been demonstrated amongst others by Kemsky [26]. Unfortunately soil gas radon measurements were not available in the area we considered, hence we have not taken this into account in the analysis.

The paper is organized as follows. In the next section, the dataset is introduced. A brief review of KED and GLS spatial regression is provided in Section 3 along with the details of our implementation. Section 4 shows the main results of this study, whereas the discussion in Section 5 concludes the paper.

## 2. Experimental Section: The Data

**Figure 1.**
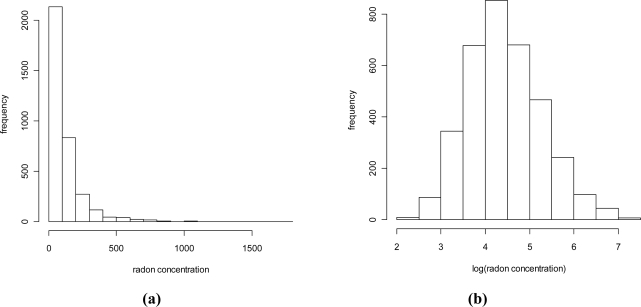
Histogram of IRC on **(a)** natural and **(b)** log scale.

The data considered in the present study were collected within an indoor radon gas monitoring survey conducted by the Agency for Environmental Protection (ARPA) of the Lombardy Region during 2003–2005, aimed at mapping radon indoor concentrations (IRC) in its regional territory. To carry out the survey, the region was divided into two parts, according to the morphology and bedrock types. The area with hills or mountains was investigated more intensively compared to the plain, since a higher variability in radon concentration distribution can be expected due to the geological and morphological characteristics. Measurements were performed in 3,512 buildings (working places or dwellings) located on the ground floor with the necessary characteristics to ensure the tests were representative and comparable. These measurements were carried out using a CR-39 trace detector, positioned in-situ between the end of September and the beginning of November 2003. The detectors were changed after six months and the two semester measures were recorded. The annual average values are considered in this paper. The mean value of IRC is 124 Bq/m^3^ ranging from 9 Bq/m^3^ to 1,796 Bq/m^3^. Table 1 shows some summary statistics of the IRC distribution in Lombardy while Figure 1(a) shows the histogram of IRC.

This histogram shows a clear asymmetry. This feature has been observed in many studies [12]. Nero and colleagues (1986) demonstrated empirically that indoor radon measurements could be well modelled using the log-normal distribution. Figure 1(b) shows the histogram of IRC taken on the log scale which looks reasonably Gaussian supporting the log-gaussianity assumption. Assuming that the radon concentrations taken on the log scale depend on a number of (roughly equally randomly distributed) factors through an additive model, log-gaussianity was motivated by the central limit theorem [20]. Many authors, however, discussed this issue investigating alternative assumptions, in particular see [30–32].

**Figure 2.**
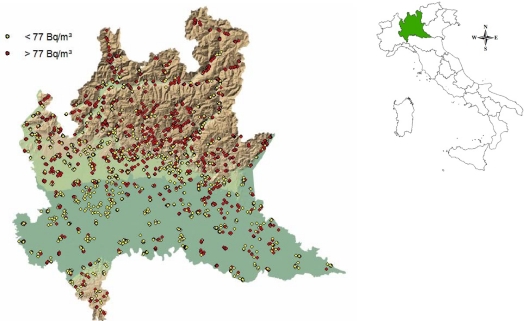
The study region: measurement point locations (indoor radon regional survey 2003–2004) classified according to whether the measured value is above or below the sample median.

**Figure 3.**
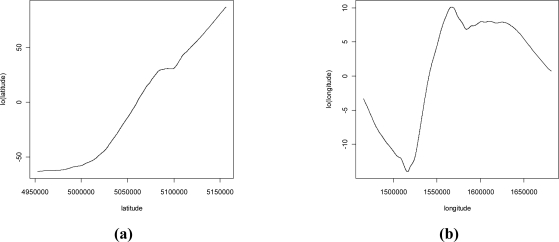
**(a)** South-North trend of IRC. **(b)** East-West trend of IRC.

Figure 2 shows the 3,512 measurement points considered in this paper. Higher IRC values tend to cluster on the territory and concentrate in the Alps area in the north of the region whereas lower values characterise the southern plain. The south-north trend is also evident in Figure 3 where spatial trends of IRC with respect to latitude (a) and longitude (b) are displayed. The trend is estimated non-parametrically by using a generalized additive model with LOESS specification of the coordinate’s effect [33]. The West-East trend appears somewhat negligible.

In the remaining part of this section, we consider some factors which can potentially affect IRC and whose effects will be discussed in detail in the following sections. We briefly describe the data sources and how these variables were coded for modelling hereafter. First the geogenic secondary variables are considered while building characteristics are described in subsection 2.2.

### 2.1. Geogenic Factors

The geologic features which may explain the spatial variation of IRC, concern proprieties like radioactivity content in bedrock, the tectonic framework and soil permeability. These proprieties, affecting the quantity of generated radon and how it flows from the earth, can be derived from lithological and soil maps. Using a geological classification, which is closely related to the geographical scale of the phenomenon under consideration, is extremely important since it has been shown that the proportion of variability that can be attributed to geological units increases with the level of detail of the geological information [34,35].

An important aspect regards the classifications of lithologies with respect to their potential content of radioactivity [36]. Some lithological classes, such as acid volcanic or gneiss, have higher radioactive contents. Other lithologies, as limestone and dolomitic rock, have a smaller content of radium hence their radon emanation is expected to be lower. However in weathered carbonate rocks like dolomite, radon emanation is generally high in spite of low bulk uranium concentration. As suggested by a referee, a reason for this might be that residual clay coatings at fractures and solution cavities absorb radium and have thus a high radon emanation power from large internal surfaces. This fact may explain the observed high IRC-values above dolomite observed in our data (see Table 2).

Furthermore, high IRC can be found in areas with low levels of radium too, especially in those areas characterized by fractured rocks or intensive tectonic framework. This may occur since the presence of different types of faults (normal, reverse or strike-slip) and regional thrust fault allows for the migration of the gas from deeper origins favouring its entry into homes [0,38]. Although the alluvial plain is usually described as a single geo-lithological unit, it is useful then to differentiate areas according to general characteristics of permeability as high emissions of radon are more likely to be found in permeable soils, *i.e.*, sandy or gravelly soils of the foothill area, whereas fine soils are a natural barrier to the upwards movement of the gas. In this way, alluvial deposits can be identified using the soil map. This has the advantage of describing the peculiarity of the subsoil at immediate contact with the basement of buildings where factors inducing the transport of radon from the soil into houses, *i.e.*, convective flow, are expected to be more important.

In order to derive secondary information which accounts for all the abovementioned geological features for the whole region, we merged the information of a geologic and a soil map both at the scale 1:250,000 [39] by a GIS exercise. More specifically, the geological map was used for the Alpine area, grouping the original lithologies (more than 100 Geological units) into seven classes namely:

- acid rocks: igneous and metamorphic rocks like rhiolite, granites, gneiss and orthogneisses
- basic rocks: igneous and metamorphic rocks like andesite, diorite, amphibolite and serpentinite
- metamorphic rocks: phyllite, schists and mica schists
- dolomite rocks
- limestone
- alluvial fan: fan-shaped deposits at the exit of main valley
- debris: landslides, rock falls and shallow debris flows, due to action of gravity.

The soil map was used to partition the alluvial plain into the following four classes:

- alluvial plain from mountain valley: deposits of fluvial rivers forming floodplains and terraces of river valleys, in the mountain area
- moraine: accumulation of unconsolidated glacial debris (soil and rock)
- foothill deposit: deposits of fluvial rivers, highly permeable, in the transition zone between plains and low relief hills to the adjacent topographically high mountains
- alluvial plain: deposits of fluvial rivers, low permeable, in the Po valley.

**Figure 4.**
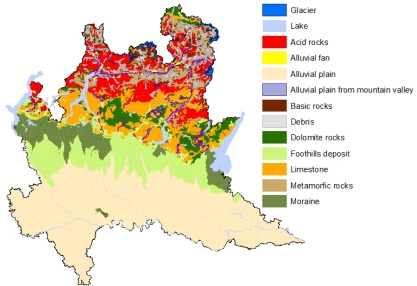
Geological types of Lombardy.

The obtained geological map, consisting of eleven geological types or classes, is reported in Figure 4. Subsequently, each sampling point was assigned to one of the eleven types by linking the sample locations to the geological map [40]. Table 2 shows some summary statistics of IRC by geological classes. Geological classes with the highest IRC are Debris, Dolomite and Acid rocks. The geologic typologies, as coded above, are secondary information mostly aiming at grasping large scale spatial variation; however these typologies are a poor measure of local behaviour. As mentioned above, geology can also act at a lower scale mostly through the tectonic framework as it can be expected that high IRC levels are found close to tectonic lineaments since cracks and holes can foster the gas to flow upwards from deeper origins. In order to account for this local geological component, we considered the distance of each sample location to the closest tectonic lineaments.

**Figure 5.**
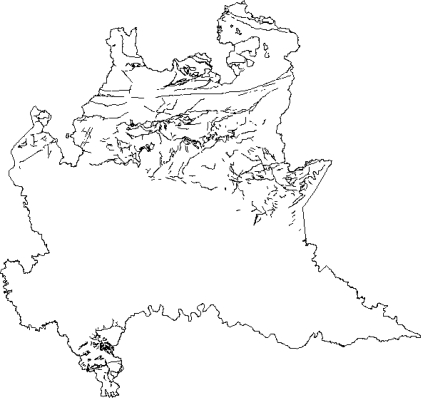
Tectonic framework in Lombardy.

This information was obtained from the map of tectonic lineaments of the Lombardy region (see Figure 5) [39]. Sample locations were superimposed to this map and the distance of each point *s* to the lineaments (coded as vectorised lines) were calculated. Using *A* to indicate a tectonic line, the distance of interest was calculated as [41] $d(s,\;A)=\underset{x\in A}{\text{inf}}\left\|s-x\right\|$.

### 2.2. Building Factors

In the regional survey, the principal characteristics of the buildings, included in the sample, were obtained by means of a questionnaire administered to dwellers. We focused on those factors which were expected to affect the IRC. Table 3 shows summary statistics of IRC as a function of the building characteristics considered in the rest of the paper. All of them seem to have a relevant effect on IRC. The average concentration is about 24 Bq/m^3^ higher for single buildings (*i.e.*, detached house or any building that is completely separated on all sides from any other structures) than non single (*i.e.*, terraced house, apartment block), about 35 Bq/m^3^ higher for buildings in direct contact with the ground (*i.e.*, slab on grade foundation, mat-slab foundations) than those with a basement (or crawl space), about 43 Bq/m^3^ higher for buildings with stone walls than those with walls made of other materials (*i.e.*, lateritious, hollow brick).

## 3. Methods

In this section we briefly review the methods used in this paper to assess the spatial variation of IRC and the effect of geologic and anthropogenic factors, namely kriging with external drift and GLS multivariate regression for spatial data. In what follows, *Z*(*s*) is a regionalised variable, that is to say, a variable which is measurable at different sites of a continuously defined region D. This is understood as a trajectory of a spatial stochastic process defined on D, *i.e.*, a random field on D. This variable represents IRC measures taken on a log scale as a function of space.

We assume that that the process of interest can be decomposed as:

(1) Z(s)=μ(s)+W(s)+ɛ(s)

where *μ*(*s*) is a deterministic unknown function representing the trend of the process, whereas *W*(*s*) is an intrinsic spatial process whose variogram is indicated by 2*γ*(*h*) = Var(*W*(*s* + *h*) – *W*(*s*)) and *ɛ*(*s*) is a random noise component representing an unstructured source of spatial variability. The variogram is the main tool used in Geostatistics to account for the degree of spatial continuity of a regionalized variable as a function of the separation distance and direction.

### 3.1. Kriging with External Drift

Kriging is a general approach for stochastic spatial interpolation in which the continuous regionalized variable of interest *Z*(*s*), sometimes referred to as the primary variable, is predicted at any unsampled location *s* of the study area D using the values of *Z* measured at different locations, *Z*(*s_i_*), *i* = 1, …, *n* ([7,42]). The predicted value at location *s*, *Z̄*(*s*), is calculated as an affine linear combination of the *n* observed values in such a way that it is a BLUP predictor *i.e.*, the best linear unbiased predictor. The coefficients of the linear combination, which multiply each measure *Z*(*s*_i_), denoted by *α*_1_, …, *α_n_* hereafter, are called the kriging weights. Unbiasness means that the predictor satisfies *E*(*Z̄*(*s*) – *Z*(*s*))= 0 whereas the adjective “best” means that the kriging has the minimum mean squared error amongst linear and unbiased predictors *i.e.*, it minimises *Var*(*Z̄* (*s*) – *Z*(*s*)) = *E* (*Z̄*(*s*) – *Z*(*s*))^2^. The kriging weights depend upon: (i) the spatial configuration of the data, (ii) the location of the prediction relative to the data locations and (iii) the spatial dependency of the process as measured by the variogram. The unbiasness condition above is guaranteed by imposing a set of constraints depending on the particular kriging predictors and the predictor is derived using the ordinary Lagrange method. Different kriging typologies depend on the hypothesised mean function. Ordinary kriging refers to a spatial process which is mean stationary, whereas universal kriging refers to a trend surface which is a function of site coordinates. Kriging with an external drift (KED) [43] is a variant of kriging that accounts for the spatial trend of the regionalised primary variable by using exhaustive secondary information provided by spatial covariates *X*(*s*) *i.e.*,:

(2) $$\mu (s)=\beta^{\prime }X(s)$$

where *β* is a column vector of unknown coefficients. More specifically, in the following section we considered a specification of KED where the spatial covariate is a categorical variable *i.e.*, a variable that takes a finite number of unordered modalities. In particular, we used the geological classification described in the previous section to specify the drift. In order to implement KED, we constructed a set of dummy variables *i.e.*, binary variables, *I*_g_ defined as *I_g_*(*s*) = 1 if *s* ∈ *g* and *I_g_*(*s*) = 0 otherwise, where *g* is one of the geologic categories defined above. Provided that the model includes an intercept, ten dummies suffice to encode this geologic factor. We note that, although some authors suggest that the regional covariates used in KED should change smoothly in space, nothing prevents one from using a dummy or a categorical covariate (see [7], pp. 356–357 and references therein). We also observe that, when a categorical variable coded as a set of, say, *k* – 1 variables *I_g_* as the one discussed above, is included in the drift, the unbiasness condition above requires a set of *k* constraints *i.e.*, one for each category of the secondary variable. More specifically, in addition to the usual kriging constraint, requiring that the kriging weights sum to 1, which descends from the inclusion of an intercept in the regression, there are other *k* – 1 constraints $\sum_{i=1}^{n}\alpha_{i}I_{g}(s_{i})=I_{g}(s)$, *g* = 1, …, *k* − 1. In other words the kriging weights pertinent to the measurement point *s_i_* falling into the same category to which the prediction point *s* belongs, sum 1, whereas the sum of weights pertinent to the other points must be 0.

As it has been observed [35], to a large degree, the distinction between the systematic and the structured random part of the Formula (1) is arbitrary and also depends on the resolution of the covariate used in the deterministic part, *i.e.*, the geological classes in the present application, the variability due to inhomogeneity of IRC within geological units at the chosen resolution (apart from measurement uncertainty) and the misclassification of geologic substratum. The higher the resolution and the more distinct the geological units, the better one could expect that IRC is predictable by the systematic component, whereas when different geological structures mix up in different classes as a consequence of a low resolution, the spatial process might be largely influenced by the random fluctuation representing the inhomogeneity of the geological units. We also note that the implementation of this method requires knowing exhaustively the secondary variable since, as explained elsewhere in this paper, drawing a map necessitates a prediction step of the kriging algorithm on a grid of points for which the covariate values must be available. Thus, the quality of the prediction depends on the quality of the secondary variable field.

When pollutant maps are constructed, relevant information is provided by probability maps, that is to say, maps which represent the probability of getting a pollutant concentration greater than a pre-assigned value *τ*. This threshold may be related to health considerations *i.e.*, a value of the pollutant that is somehow known to be dangerous for human or animal health or to a “level of action” *i.e.*, a value above which some interventions have to be undertaken or planned. It can be noted that the Italian legislation does not define IRC action levels explicitly. In many circumstances, the values suggested by the 90/143/Euratom recommendation are adopted. The Euratom recommendation suggests 200 and 400 Bq/m^3^ (about the 85th and 96th percentile of our sample) for, respectively, the future construction standard and for considering remedial interventions in existing buildings. Subsequently, we considered these two levels to identify the area of the region more prone to high IRC. The approach used for this aim is based on the conditional (direct) Monte Carlo simulations [7]. Assuming the IRC process to be log-gaussian, we simulated a large number of independent maps, say *B*, on the prediction grid of points according to Cholevsky’s decomposition method of the spatial covariance matrix. The proportion of the simulated values $Z_{b}^{*}(u)$, *b* = 1, …, *B* that fell above *τ* out of the *B* simulations for each point *u* of the prediction grid, *i.e.*, $p(u)=\frac{1}{B}\sum_{b=1}^{1}I(Z_{b}^{*}(u)>\tau )$, is then a consistent estimate of the probability of interest.

### 3.2. GLS Multiple Regression

In order to assess the potential effect of geological and building factors on the IRC, a multiple linear regression model has been adopted. Let *Z* be the vector of measures of the regionalised variable taken at the *n* sample sites *s_i_ i* = 1, …, *n*, and *X* a *n* × *p* matrix of *p* predictor variables also measured at these locations. The multiple linear regression model is given by:

(3) Z=Xβ+ɛ

where *β* is a (*p* × 1) vector of unknown parameters and *ɛ* is a vector of error terms with zero mean and variance-covariance matrix ∑(*θ*). Assuming *θ* being known, the generalized least squares (GLS) estimator of *β* is given by:

(4) $${\hat{\beta }}_{\text{gls}}={\left(X^{\prime }\Sigma {\left(\theta \right)}^{-1}X\right)}^{-1}X^{\prime }\Sigma {\left(\theta \right)}^{-1}Z$$

Usually the covariance parameters are unknown and have to be estimated. Indicating by *θ̄* an estimate of the covariance parameters *θ*, estimable GLS of *β* are given by:

(5) $${\hat{\beta }}_{\text{egls}}={\left(X^{\prime }\Sigma {\left(\hat{\theta }\right)}^{-1}X\right)}^{-1}X^{\prime }\Sigma {\left(\hat{\theta }\right)}^{-1}Z$$

Schabenberger and Gotway ([44] pp. 256–259) proposed an iteratively reweighted generalized least squares (IRWGLS) algorithm to obtain simultaneous estimation for *β* and *θ*. This algorithm is articulated in the followings steps:

1. obtain a starting estimate of *β*, *β̄*_0_ that does not depend on *θ* (for example by OLS)
2. compute the residuals *r*(*s*) = *Z*(*s*) – *X* (*s*) *β̄*
3. estimate the variogram of the residuals, obtaining an estimate of *θ*, *θ̄*
4. obtain a new estimate of *β* using Equation (5)
5. Repeat steps 2–4 until the relative change in estimates of *β* and *θ* are small.

As stopping rule we use $\text{max}\left\{\delta_{j}^{\beta },\;\delta_{k}^{\theta }\right\}<10^{-6}$ in step 5 where:

$$\delta_{j}^{\beta }=\frac{\left|{\hat{\beta }}_{j}^{u}-{\hat{\beta }}_{j}^{u+1}\right|}{0.5\left(\left|{\hat{\beta }}_{j}^{u}\right|+\left|{\hat{\beta }}_{j}^{u+1}\right|\right)}\;\text{and}\;\delta_{k}^{\theta }=\frac{\left|{\hat{\theta }}_{j}^{u}-{\hat{\theta }}_{j}^{u+1}\right|}{0.5\left(\left|{\hat{\theta }}_{j}^{u}\right|+\left|{\hat{\theta }}_{j}^{u+1}\right|\right)}\;$$

with ${\hat{\beta }}_{j}^{u}$ and ${\hat{\beta }}_{j}^{u+1}$ ( ${\hat{\theta }}_{j}^{u}$ and ${\hat{\theta }}_{j}^{u+1}$ respectively) the estimates of *β* (*θ*) obtained from two successive iterations.

## 4. Results

### 4.1. IRC Mapping

**Figure 6.**
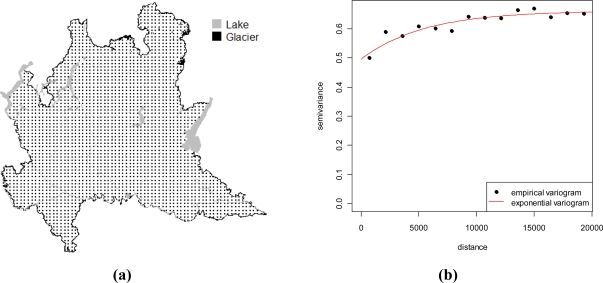
**(a)** Grid used for prediction. **(b)** Estimated variogram.

**Figure 7.**
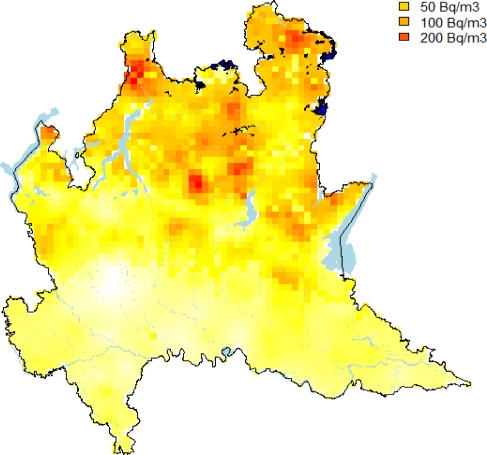
Maps of predicted IRC.

In order to produce maps representing IRC on the regional territory, KED and conditional simulations have been adopted. As mentioned above, geological classes were used as a secondary variable in KED for spatial prediction. To produce maps of IRC, a dense regular grid of 2,515 prediction points covering the Lombardy territory was preliminarily built. Figure 6(a) shows the grid used to produce the maps. To derive the geological class necessary for KED prediction, this grid was superimposed to the geological map reported in Figure 4 by a spatial join in a GIS environment. Table 4 shows the number of prediction points by geological classes. As far as the implementation of the kriging prediction is concerned, this requires knowing the variogram which has to be estimated on the data. To this end we detrendized the data by estimating the mean function (2) via OLS first and then estimating the variogram of the residuals. A weighted least square estimate of an isotropic exponential variogram was used for this, see Figure 6(b). Figure 7 shows the surface of IRC as estimated by KED. To evaluate the performance of the prediction method, we adopted a one-leave-out cross-validation technique, removing one sample observation at a time and predicting this observation by the rest of the sample. Although we have not reported the results in detail, we here mention that the cross-validation residuals $r_{i}^{\text{KED}}=Z(s_{i})-Z^{*}(s_{\left[i\right]})$ showed a reasonably good performance of KED since their average was close to zero and their histogram symmetrically shaped. A good correlation between observed and predicted value was also found.

**Figure 8.**
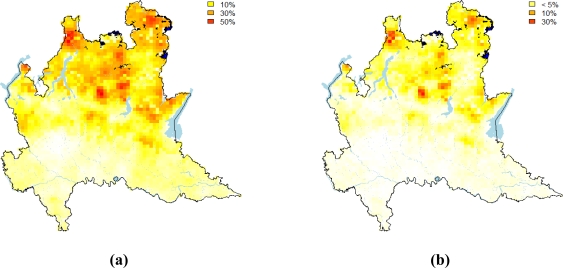
**(a)** Probability maps for the reference values of 200 Bq/m^3^. **(b)** Probability maps for 400 Bq/m^3^.

Using the model obtained by KED, we simulated 1,000 maps of log IRC on the grid of Figure 6(a) and after an exponential transformation, we calculated the proportion of simulated values falling above the two levels as suggested by the Euratom recommendation. Maps reported in Figures 8(a) and (b) show the results obtained by this analysis. They show that smaller values of IRC and the low probability of falling above the thresholds considered tend to occur in alluvial plain and moraine. Other geological structures seem to be correlated much more with large values of IRC. In particularly, we noted large values of IRC along the transition zone between the alluvial plain and the mountain area. This is the area of foothill deposits which are highly permeable. Hence the maps suggest a clear tendency of large IRC to be higher moving towards the northern part of the region close to the Alps, due to the presence of volcanic rock and intensive fractured dolomite rock.

### 4.2. Assessing Anthropogenic and Geologic Influential Factors for IRC

**Figure 9.**
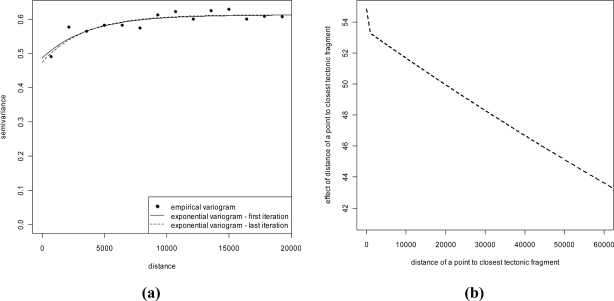
**(a)** Exponential semi-variograms related to first and last iteration of IRWGLS algorithm. **(b)** Effect of the distance of a point to the closest tectonic lineament.

In order to evaluate the effect of building characteristics and geologic factors on IRC, we applied the methodology described in Section 3.2 which was implemented by an R code [45]. The GLS regression includes the explicative variables described in section 2, namely whether the building is in direct contact with the ground, whether it is a single unit and whether it has stone walls, the geologic type of the soil and the distance from the closest tectonic lineament. Since the latter was expected to have a non linear effect on IRC, it was entered in the model through a linear B-spline [46]. Using a B-spline transform for the distance from the nearest tectonic fault seems a natural solution given the approach followed in this paper. In fact, in addition to increasing model flexibility, it is also computationally convenient as it amounts to extend the set of the explanatory variables of the regression by adding the value of the basis function evaluated at knots. With this expanded set of covariates, the regression can be fitted in the least squares framework, described in the previous section. The model considered is then a semi-parametric one which preserves the additive nature of the predictor. We adopted an exponential specification for the variogram. The IRWGLS algorithm converged reasonably quickly, after 5 iterations. The estimated variograms, after the first and last iteration, are reported in Figure 9(a), which shows that only moderate adjustments occurred during the procedure.

The regression coefficients estimated via IRWGLS are reported in Table 5. Significant effects were found for all the building parameters and for many geological classes.

We used only one node in the linear B-spline as a number of preliminary explorative analyses suggested the presence of one potential change point in the covariate effect. The node was located at 1,000 m. Our hypothesis was that only those buildings close to a lineament can be influenced by a more irregular soil, *i.e.*, more ground cracks and faults, which can facilitate the gas stemming into a building. In fact we re-estimated the model for a number of different locations of the node and found that the change of slope gets less and less pronounced as the node moves away from zero. In addition, the significance of the correspondent spline basis component tends to decline. Both, statistic significance and the slope difference between the two linear pieces, disappear as the node gets as large as about 4,000 m. The effect of the distance is depicted in Figure 9(b).

**Figure 10.**
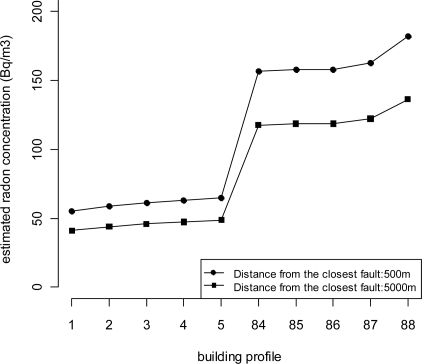
Building profiles with the lowest and highest estimated IRC.

Finally, we classified the different typologies of buildings according to their proneness to IRC on the basis of the expected value of IRC, as estimated by the regression model. The combination of building factors and geologic classes identifies 2 × 2 × 2 × 11 = 88 different building profiles assuming the distance from the closest tectonic lineament fixed to any predefined value. This can be done without loss of generality, given the additive nature of the model. These profiles are ranked from the least to the most prone to IRC. We have not reported the whole list of ranked profiles here, but we have summarised the results in Figure 10 where the top and bottom five profiles are reported for two values of the distance from the closest tectonic fault namely 500 m and 5,000 m [48].

The last five groups, *i.e.*, most prone to high IRC, represent buildings that are in contact with the ground and are made of stone walls. All but one, profile number 87, are single buildings. The geologic class of profiles 87 and 88 is Debris, whereas the geologic class of profiles 84, 85 and 86 are, respectively Dolomite rocks, Acid rocks and Alluvial plain from mountain valley. The top five profiles are not in contact with the ground, all but profile 3 are not single buildings and all but profile 5 have no stone-walls. The geologic class of these profiles are Alluvial Plain (profiles 1, 3, 5), Basic Rocks (2) and Moraine (5). Finally, an increase in IRC can be expected to be found for those buildings which were constructed closer to tectonic lineaments and this effect appears to be more pronounced for more prone profiles. This analysis, hence, shows why some building typologies are particularly exposed to high IRC whereas others tend to protect people living inside them. Furthermore, high IRC can be expected in buildings built on a particular geologic environment.

## 5. Discussion and Conclusions

Air pressure inside the buildings is usually lower than that in the surrounding soil. Because of this difference in pressure, the radon gas can enter rooms through foundation cracks and other openings in the building structures. The indoor concentrations depend on building characteristics as well as geologic factors. These two aspects deeply interact particularly in a large and inhomogeneous region as the one considered here.

The results obtained in this paper are two-fold. On one hand, by means of maps of the Lombardy region, we depicted the areas more prone to high IRC. We found that these areas are mostly concentrated on particular geologic structures of the ground. On the other hand, we indicated some possible levers represented by architectonic characteristics of the building which can be employed to efficiently protect the population.

Concerning the former point, it is known that IRC is spatially structured to some extent; hence we used a geostatistical approach to draw the maps. In particular, we used georeferenced data collected within an indoor radon gas monitoring survey conducted by the Agency of Environmental Protection of Lombardy Region (Italy) in 2003. It is commonly argued that the geologic nature of the ground is relevant information for the map, since the ground is the main origin of natural radioactivity. To account for this potential source of regularity, we linked the data with external spatial databases, containing information about the lithology and the soil, to identify sensible geological structures. The rational of our model is that even though the geologic structure can be relevant, other unmeasured spatially structured components may influence IRC. Hence, we did not expect that this secondary information suffices to explain the spatial variation of the phenomenon under study as it seems confirmed by the variogram in Figure 6(b), which measures spatial regularity after removing the effect of geological classes. In addition to this, the resolution of the geological information can play a crucial role in the predictive capability of this secondary variable since it can be reasonably argued that when different geological structures mix up in different classes as a consequence of a low resolution, spatial regularity may remain in the data even after the geological variability has been controlled. For this reason, we based our maps on a kriging with an external trend procedure. The maps confirm that there is substantial agreement among the areas found to be more prone to high IRC and the geological structures where higher concentrations could have been expected in advance. Had one been interested in the impact that IRC has on the population, the spatial distribution of inhabitants should have been taken into account since the northern part of the region, the one more exposed to high IRC, is largely mountainous and less populated. A more precise evaluation of this is beyond the scope of this paper and is a matter for future research.

It is also known, however, that lithology and soil porosity are not the only relevant factors affecting IRC. IRC is a composite phenomenon, influenced to some extent, by other environmental and anthropogenic factors. Moreover, geologic classes are mostly large scale information whereas local geologic components can be crucial too. In particular, we considered the tectonic structure of the ground. Our second aim was then to evaluate the effect of all these potential factors simultaneously through a multiple regression model. Building information was collected within the survey by administering a questionnaire to the dwellers, whereas information on tectonic lineaments and geology was obtained by linking tectonic, soil and lithologic maps. We accounted for the spatial correlation in the data by using an iteratively reweighted generalized least squares algorithm. Our analysis allowed us to rank different building profiles, identified by architectonic characteristics as well as the geologic nature of the ground, according to their proneness to IRC. Hence, we showed why some building typologies are particularly exposed to high IRC whereas others tend to protect the people living inside them. This analysis also pointed out that the beneficial effect of these instruments can be extremely relevant in those areas that have a geologic nature richer in uranium and uranium progeny and where the ground structure is more fragmented and characterized by relevant permeability. In particular we provided an estimate of the effect of the building distance to the nearest tectonic fault on IRC and found that buildings in a close proximity of a lineament are more exposed to high IRC. Although the tectonic structure has already been argued as a mechanism which facilitates radon to flow on a qualitative ground, to the best of our knowledge, this paper, is the first to attempt to quantify it. We agree that the geologic information we have, does not have a high spatial resolution. However, more detailed maps are currently not available for the entire areas we are interested in. It will be interesting to further investigate the geologic effects and to better understand the impact of this potential determinant of indoor radon accumulation, perhaps concentrating the analysis on smaller portions of the territory where more detailed spatial information is likely to be available.

Hence, the results of this paper may suggest the opportunity to differentiate construction requirements in a large and inhomogeneous area according to different places. Furthermore, when considering buildings already present in the territory, our findings may help agencies, involved in environmental radioprotection, to identify what type of dwellings should be monitored more carefully and which parameter it would be best to intervene on in order to reduce IRC when necessary. Hence, this paper suggests a way of estimating the effect a given remedial action can have in terms of IRC reduction, which is a preliminary step in order to evaluate its cost/benefit ratio.

## Figures and Tables

**Table 1.** Summary statistics of IRC in Lombardy.

**Statistics**	**Value**
No. of sites	3,512
Mean	124
Median	77
Standard deviation	141
Min	9
Max	1,796

**Table 2.** Summary statistics of indoor radon concentration by geological classes.

**Geological classes**	**No. of sites (%)**	**Mean Bq/m^3^**	**Sd Bq/m^3^**	**Median Bq/m^3^**	**% above 200 Bq/m^3^**	**% above 400 Bq/m^3^**
Alluvial plain	833 (24%)	66	50	53	2	0
Foothill deposit	667 (19%)	118	115	76	15	3
Limestone	333 (10%)	137	148	88	18	6
Alluvial fan	136 (4%)	125	115	88	16	3
Debris	246 (7%)	207	224	148	33	9
Dolomite rocks	246 (7%)	198	197	127	30	13
Acid rocks	183 (5%)	192	204	113	29	13
Basic rocks	32 (1%)	83	82	59	6	3
Metamorphic rocks	174 (5%)	148	125	111	22	3
Alluvial plain from mountain valley	213 (6%)	168	169	117	28	8
Moraine	437 (12%)	92	90	64	9	2

**Table 3.** Summary statistics of indoor radon concentration by building characteristics.

**Building characteristics**	**No. of sites (%)**	**Mean Bq/m^3^**	**Sd Bq/m^3^**	**Median Bq/m^3^**	**% above 200 Bq/m^3^**	**% above 400 Bq/m^3^**
**Type of building**						
Single	2,364 (67%)	131	140	84	18	5
Non single	1,073 (31%)	107	136	68	10	3
Missing value	75 (2%)					

**Type of soil connection**						
Contact with ground	1,259 (36%)	146	156	91	21	7
Basement/Crawl space	2,116 (60%)	111	130	71	12	3
Missing value	137 (4%)					

**Wall material**						
Stone	511 (15%)	161	186	104	24	7
Other materials	2,946 (84%)	118	131	74	14	4
Missing value	55 (1%)					

**Table 4.** Number of grid points by geological classes.

**Geological Classes**	**No. of grid points**
Alluvial plain	1,030
Foothill deposit	316
Limestone	212
Alluvial fan	8
Debris	102
Dolomite rocks	175
Acid rocks	233
Basic rocks	38
Metamorphic rocks	180
Alluvial plain from mountain valley	33
Moraine	188

**Table 5.** Estimated coefficients of GLS model. Alluvial plain is the geologic baseline category [47].

	**Coefficients**	**Standard error**	**t-value**	**p-value**
Intercept	4,004	0,101	39,834	<0,0001
Foothill deposit	0,357	0,074	4,819	<0,0001
Limestone	0,355	0,092	3,875	0,0001
Alluvial fan	0,412	0,116	3,559	0,0004
Debris	0,732	0,102	7,203	<0,0001
Dolomite rocks	0,584	0,101	5,789	<0,0001
Acid Rocks	0,592	0,110	5,388	<0,0001
Basic rocks	0,066	0,178	0,372	0,7102
Metamorphic rocks	0,575	0,113	5,105	<0,0001
Alluvial plain from mountain valley	0,592	0,102	5,778	<0,0001
Moraine	0,140	0,088	1,600	0,1096
Single building	0,110	0,030	3,704	0,0002
Contact with ground	0,189	0,029	6,494	<0,0001
Stone wall material	0,167	0,040	4,218	<0,0001
